# α2,3-sialyltransferase type I regulates migration and peritoneal dissemination of ovarian cancer cells

**DOI:** 10.18632/oncotarget.15994

**Published:** 2017-03-07

**Authors:** Kuo-Chang Wen, Pi-Lin Sung, Shie-Liang Hsieh, Yu-Ting Chou, Oscar Kuang-Sheng Lee, Cheng-Wen Wu, Peng-Hui Wang

**Affiliations:** ^1^ Department of Obstetrics and Gynecology, Taipei Veterans General Hospital, Taipei, Taiwan; ^2^ Institute of Clinical Medicine, National Yang-Ming University School of Medicine, Taipei, Taiwan; ^3^ Department of Obstetrics and Gynecology, National Yang-Ming University, Taipei, Taiwan; ^4^ Genomics Research Center, Academia Sinica, Taipei, Taiwan; ^5^ Institute of Biotechnology and Department of Medical Science, National Tsing Hua University, Hsinchu, Taiwan; ^6^ Stem Cell Research Center, National Yang-Ming University, Taipei, Taiwan; ^7^ Taipei City Hospital, Taipei, Taiwan; ^8^ Department of Medical Research, Taipei Veterans General Hospital, Taipei, Taiwan; ^9^ Institute of Biomedical Science, Academia Sinica, Taipei, Taiwan; ^10^ Department of Medical Research, China Medical University Hospital, Taichung, Taiwan

**Keywords:** α2,3-sialyltransferases type I, epidermal growth factor receptor, epithelial ovarian cancer, soyasaponin I

## Abstract

Epithelial ovarian cancer (EOC) has the highest mortality rate among gynecologic cancers due to advanced stage presentation, peritoneal dissemination, and refractory ascites at diagnosis. We investigated the role of α2,3-sialyltransferase type I (ST3GalI) by analyzing human ovarian cancer datasets and human EOC tissue arrays. We found that high expression of ST3GalI was associated with advanced stage EOC. Transwell migration and cell invasion assays showed that high ST3GalI expression enhanced migration of EOC cells. We also observed that there was a linear relation between ST3GalI expression and epidermal growth factor receptor (EGFR) signaling in EOC patients, and that high ST3GalI expression blocked the effect of EGFR inhibitors. Co-Immunoprecipitation experiments demonstrated that ST3GalI and EGFR were present in the same protein complex. Inhibition of ST3GalI using a competitive inhibitor, Soyasaponin I (SsaI), inhibited tumor cell migration and dissemination in the *in vivo* mouse model with transplanted MOSEC cells. Further, SsaI synergistically enhanced the anti-tumor effects of EGFR inhibitor on EOC cells. Our study demonstrates that ST3GalI regulates ovarian cancer cell migration and peritoneal dissemination via EGFR signaling. This suggests α2,3-linked sialylation inhibitors in combination with EGFR inhibitors could be effective agents for the treatment of EOC.

## INTRODUCTION

Epithelial ovarian cancer (EOC) is the second most common type of gynecologic cancer and a leading cause of gynecological cancer deaths in the United States. In 2016, there were 14,240 EOC related deaths and 22,280 new cases diagnosed in the United States [1]. Despite intensive treatment with cytoreductive surgery and post-operative adjuvant chemotherapy with/without anti-angiogenesis agents, EOC has the highest mortality rate among gynecological cancers because it is diagnosed in an advanced stage and has high recurrence rate [2–4]. The estimated average disease-free survival (DFS) time for EOC patients is 18 months with a 5-year overall survival (OS) rate below 30% [5]. EOC is characterized by advanced-stage presentation, multiple organ metastases, peritoneal dissemination and refractory ascites at diagnosis [6]. Currently, the diagnosis of metastasis and/or recurrence is still dependent on imaging clues and detection of carbohydrate antigen 125 (CA125) [7], both of which are limited in sensitivity and specificity. Therefore, the development of novel biomarkers is urgently required for accurate and early prediction of metastasis and treatment outcomes in EOC patients.

Sialic acids belong to a family of 9-carbon amino sugars that are widely distributed in nature as terminal sugars of oligosaccharide chains of glycoconjugates (glycoproteins and glycolipids) [8]. The sialyltransferases (ST) and sialidases regulate sialylation, which is an important posttranslational modification reported during progression of many cancers [9–11]. The ST family consists of three subfamilies with 20 anabolic enzymes [12], namely, α2,3-sialyltransferases that mediate the transfer of sialic acid with an α2,3-linkage to terminal Gal residues (ST3GalI-VI) and α2,6-sialyltransferases that mediate the transfer of sialic acid with an α2,6-linkage to terminal Gal (ST6GalI-II) or GalNAc residues (ST6GalNAcI-VI) [13, 14]. The link between the ST family and EOC was reported previously by few studies [15–17]. For example, Christie *et al* reported that sialylation of β1 integrins mediated by ST6Gal-I altered the adhesion and migration characteristics of ovarian cancer cells through the extracellular matrix leading to peritoneal metastasis [17].

In our previous study, we showed altered expression and significant increase of α2,3-linked sialylated proteins in ovarian cancer patients and the enhanced α2,3-linked sialylation was directly linked to increased expression of ST3GalI [16]. The competitive ST inhibitor, soyasaponin I (SsaI, K_i_ = 2.3μM) was shown to affect CMP-Neu5Ac binding to ST, but did not inhibit other glycosyltransferases and glycosidases [18]. Further, SsaI inhibited α2,3-linked sialic acid expression in B16F10 melanoma and MDA-MB-231 breast cancer cell lines that resulted in increased adhesion and decreased migration and invasiveness of the two cell lines [19, 20].

Epidermal growth factor receptor (EGFR), also known as ErbB-1 or HER1, is a transmembrane receptor tyrosine kinase (RTK) and a member of the human epidermal receptor (HER) family, which is involved in many cell signaling pathways. EGFR is overexpressed in many cancers and regulates cancer invasion, metastasis, and angiogenesis [21–25]. After binding to specific ligands (EGF or TGF-α), EGFR undergoes conformation changes and forms homo- or hetero-dimers with other HER family members [26–31]. After autophosphorylation, the dimeric EGFR recruits and activates various downstream cytoplasmic and nuclear signaling proteins, which regulate multiple cellular processes, including proliferation, migration, differentiation, survival, and apoptosis [26–28]. Overexpressed EGFR is associated with poor prognosis in ovarian cancers [32–34]. Although EGFR is an attractive therapeutic target, clinical trials with several EGFR inhibitors have demonstrated modest anti-tumor effects on ovarian cancer [34–36]. Therefore, in this study, we investigated the prognostic value of ST3GalI and its relationship with EGFR signaling in ovarian cancer using both *in vitro* and *in vivo* models including human ovarian cancer patient microarray datasets.

## RESULTS

### ST3GalI is a prognostic factor for migration and peritoneal dissemination of human ovarian cancer cells

**Figure 1:**
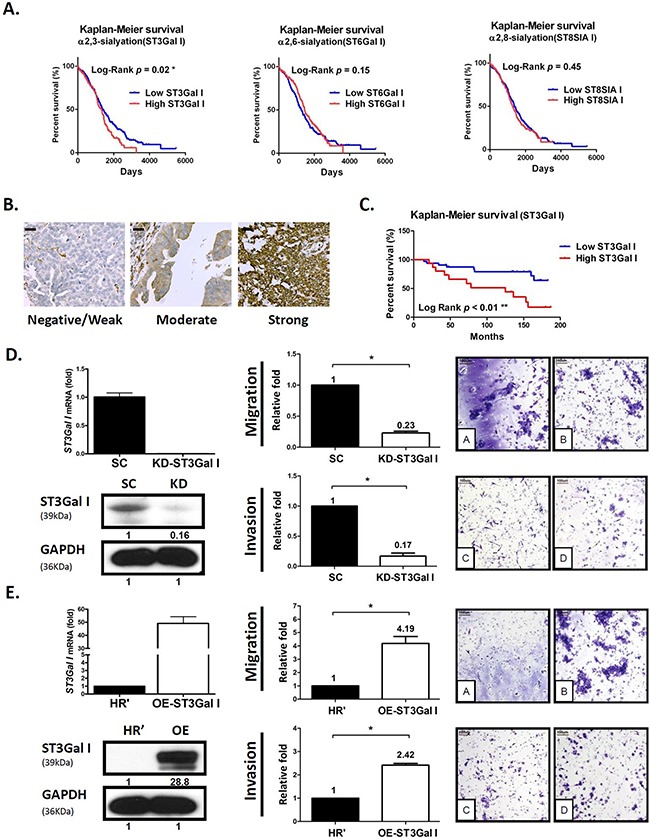
ST3GalI is a prognostic factor for tumor migration and peritoneal dissemination of human ovarian cancer **(A)** Using Oncomine TCGA ovarian cancer genomics (562 ovarian carcinoma samples analyzed on an Affymetrix Human Genome U133 array; 12,624 measured genes), we compared different ST mRNAs, including α2,3-, α2,6-, and α2,8-linked ST, with survival time using a tercile approach. Patients with an upper one-third mRNA expression were defined as the high subgroup, while others with lower two-thirds mRNA expression were defined as the low subgroup. **(B-C)** IHC analysis of ST3GalI was performed on commercial human ovarian cancer tissue array samples (Super Bio Chips, CJ2, Korea). The intensity scores were as follows: 0, no staining; 1, weak; 2, moderate; 3, strong. Low ST3GalI included weak, moderate or no staining; high ST3GalI was defined as strong staining. Scale bars representing 20μm were added from an image taken at identical magnification and resolution. The percentage was determined in the early stage (FIGO stage I &II) or late stage (FIGO stage III&IV) disease groups. The Fisher's exact test was used to statistically analyze the percentage for the early and late stages. Kaplan-Meier survival curves were used to analyze OS in low- and high-ST3GalIgroups. **(D-E)** Transwell migration and matrigel invasion of ES2 human ovarian cancer cells with either ST3GalI knocked-down or over-expressed was assayed. Total numbers of cells in 7 random fields were counted. Data shown are the mean ± SD of 3 separate experiments (*: *p*< 0.05, **: *p*<0.01.). Data on the Y-axis represented relative value compared to control. Immunoblots were quantified using Image J software.

First, we analyzed the correlation between overall survival (OS) rate and expression data of sialyltransferases (high, moderate or low) using the Human Genome U133A Array (562 tumor cases) available from The Cancer Genome Atlas (TCGA) at the Oncomine website. We observed that ST3GalI played a more critical role in disease progression than ST6GalI (α2,6-sialyltransferase) and ST8SIAI (α2,8-sialyltransferase). Kaplan-Meier analyses of TCGA cohort specimens showed that EOC patients with high ST3GalI expressing tumors demonstrated poor survival rates (Figure 1A and Table 1). Furthermore, immunohistochemical (IHC) staining using the human EOC tissue array (CJ2 provided by SUPER BIO CHIPS, Seoul/South Korea) showed that higher intensity staining of ST3GalI (Figure 1B) positively correlated with lower overall survival rate (Figure 1C). These findings demonstrated that ST3GalI had significant prognostic value in human ovarian cancer.

**Table 1:** Overall survival time in different sialyltransferases of TCGA human ovarian cancer

Sialyltransferase		Overall survival time (Days)		
		Low expression(n=376)	High expression(n=186)	*p*-value
**α2,3-sialylation**	**ST3GAL I**	1042.1±88.0	921.7±93.8	**0.02** *
	**ST3GAL II**	993.2±82.1	1020.6±114.6	0.99
	**ST3GAL IV**	969.3±81.3	1068.8±116.2	0.28
**α2,6-sialylation**	**ST3GAL V**	1005.1±83.1	996.4±111.5	0.38
	**ST3GAL VI**	993.9±81.3	1019.1±117.1	0.27
	**ST6GAL I**	942.7±82.7	1122.6±110.8	0.15
	**ST6GALNAC II**	955.7±77.5	1096.3±126.0	0.39
	**ST6GALNAC IV**	1016.8±84.5	972.7±106.9	0.61
	**ST6GALNAC V**	961.0±81.0	1085.6±116.6	0.62
	**ST8SIA I**	1006.1±85.8	994.5±103.1	0.45
	**ST8SIA II**	988.0±81.6	1030.9±115.8	0.22
**α2,8-sialylation**	**ST8SIA III**	979.6±82.6	1048.0±112.8	0.56
	**ST8SIA IV**	1007.8±83.3	991.0±111.0	0.49
	**ST8SIA V**	983.2±81.9	1040.8±115.2	0.86

*Kaplan Meier overall survival analysis

We further investigated the association of ST3GalI expression with EOC progression. Compared to early stages, ST3GalI expression was significantly higher in advanced stages among the various ovarian cancer datasets tested (Supplementary Figure 1A). This suggested that overexpression of ST3GalI was associated with advanced stage EOC (peritoneal seeding and distant metastases), especially in advanced serous- or clear-type cell carcinoma (Supplementary Figure 1B).

Further, we investigated the role of ST3GalI in ovarian cancer cell migration by either knocking down or overexpressing ST3GalI in human ovarian cancer ES2 cells and performing Transwell assays. We observed that downregulation of ST3GalI significantly suppressed cancer cell migration and invasion, whereas overexpression of ST3GalI enhanced cell migration and invasion (Figure 1D–1E, and Supplementary Figure 2). Interestingly, altered ST3GalI expression (knockdown or overexpression) did not significantly affect tumor growth (Supplementary Figure 3). These findings suggested that ST3GalI regulated the migration and invasiveness of ovarian tumor cells without altering tumor growth.

### ST3GalI interacts with EGFR signaling pathway in ovarian cancer

**Figure 2:**
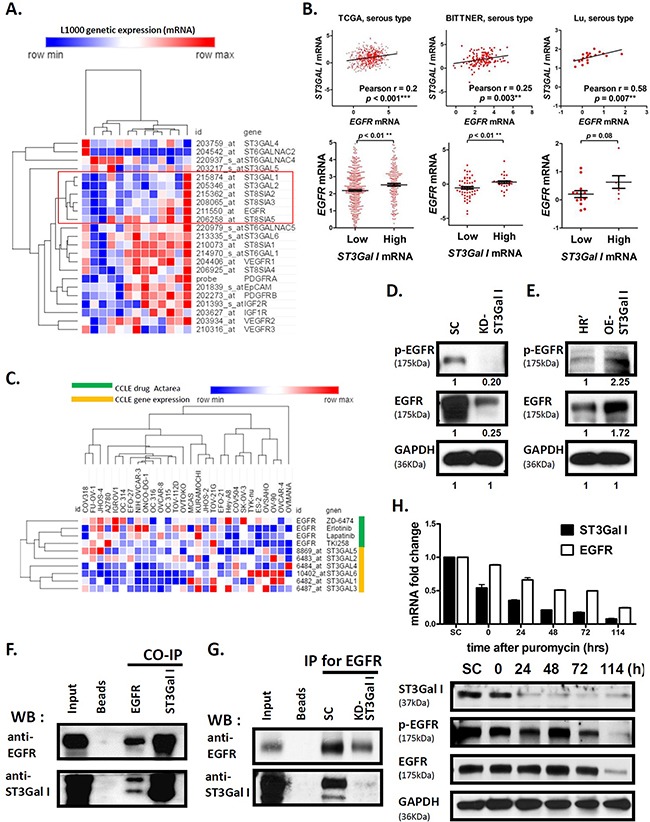
ST3GalI interacts with EGFR signaling pathway in ovarian cancer **(A)** The correlation of 20 STs and significant cell receptors in the ES2ovarian cancer cell line were analyzed by the L1000 mRNA microarray. Data shown are mean ± SD of separate repeat experiments. **(B)** The analysis of mRNA expression of ST3GalI and EGFR from Oncomine TCGA (n=562), Bittner (n=241), and Lu (n=50) ovarian cancer genomics is shown. The expression of EGFR was compared between low and high ST3GalI groups using a tercile approach. **(C)** The mRNA expression of α2,3-sialyltransferases and TKI drug efficiency (Actarea) are shown from the CCLE ovarian cancer dataset. **(D-E)** Western blot analysis of EGFR and phospho-EGFR in ST3GalI knocked-down or overexpressing cells compared to controls is shown. GAPDH was used as control (same GAPDH as in Figure 1D-1E). **(F)** The co-immunoprecipitation assay coupled with immunoblotting analysis to evaluate the protein-protein interaction of ST3GalI and EGFR is shown. **(G)** Immunoblotting of ST3Ga1I immunoprecipitated with anti-EGFR antibodies in SC and ST3GalI knock-down of ES2 cell line are shown. **(H)** Time course RNA and protein analysis showing EGFR expression during ST3GalI knock-down.

To identify the signaling pathways regulated by ST3GalI, we analyzed important cell receptors in the human ovarian cancer ES2 cell line using the L1000 mRNA microarray. Our analysis showed that ST3GalI expression was significantly associated with expression of the EGFR genes (Figure 2A). Also, the EGFR signaling pathway was overexpressed and associated with poor prognosis in more than 70% of ovarian cancer patients [32]. We further analyzed three ovarian cancer genomic datasets (TCGA, Bittner, and Lu) and found a linear relationship between ST3GalI and EGFR (Figure 2B, upper panel). We observed that ovarian cancer patients with a high ST3GalI expression demonstrated increased EGFR levels simultaneously (Figure 2B, lower panel). Further, the patients that highly expressed ST3GalI and EGFR simultaneously, demonstrated poorer clinical prognosis than others (Supplementary Figure 4A). Moreover, there was a close relationship among different subtypes of EOC such as serous carcinoma (Pearson *r* = 0.25~0.58, *p* < 0.01, Supplementary Figure 4B-4C).

Next, we analyzed the mRNA levels of the different STs, namely, ST3GalI-VI in response to EGFR inhibitor treatments (erlotinib, lapatinib, ZD-6474, TKI258) in different ovarian cancer cell lines listed in the Cancer Cell Line Encyclopedia (CCLE) database. We observed that the anti-tumor effect of EGFR inhibitors was more pronounced when the mRNA expression of STs was lower (Figure 2C, left side of heat-map). However, EGFR inhibitors demonstrated poorer anti-tumor effect in cell lines with higher expression of STs (Figure 2C, right side of heat-map). These results demonstrated synergy between ST3GalI levels and EGFR.

To understand the relationship between ST3GalI and EGFR, we investigated the EGFR expression in the ovarian cancer cells with ST3GalI either knocked down or overexpressed. We observed that ST3GalI knock down decreased EGFR expression, whereas ST3GalI overexpression enhanced EGFR levels (Figure 2D–2E). In addition, we observed that ST3GalI associated with epithelial mesenchymal transition (EMT) markers, such as E-cadherin or N-cadherin (Supplementary Figure 5) since we observed increased E-cadherin and decreased N-cadherin in the ST3GalI knock-down. Co-Immunoprecipitation analysis demonstrated that EGFR and ST3GalI were physically present in the same protein complex since ST3GalI was found in the complex immunoprecipitated from ES2 ovarian cancer cell lysates with anti-EGFR antibodies and EGFR was immunoprecipitated with anti-ST3GalI antibody (Figure 2F). Further, in the ST3GalI knock-down, we observed decreased ST3GalI immunoprecipitating with anti-EGFR antibody (Figure 2G). Interestingly, we also observed that ST3GalI regulated EGFR transcriptionally in a time-dependent manner (Figure 2H). We observed that when ST3GalI was down regulated by shRNA knockdown, an immediate and profound reduction of ST3GalI expression was followed by a stable and longer down regulation of EGFR. This finding suggested that ST3GalI may regulate the EGFR pathway via regulate factors that were upstream of EGFR in the signaling pathway or even a negative feedback mechanism. Taken together, these findings suggested that ST3GalI positively regulated EGFR.

### α2,3-sialylation inhibitor SsaI suppresses ovarian cancer cell migration and peritoneal dissemination

**Figure 3:**
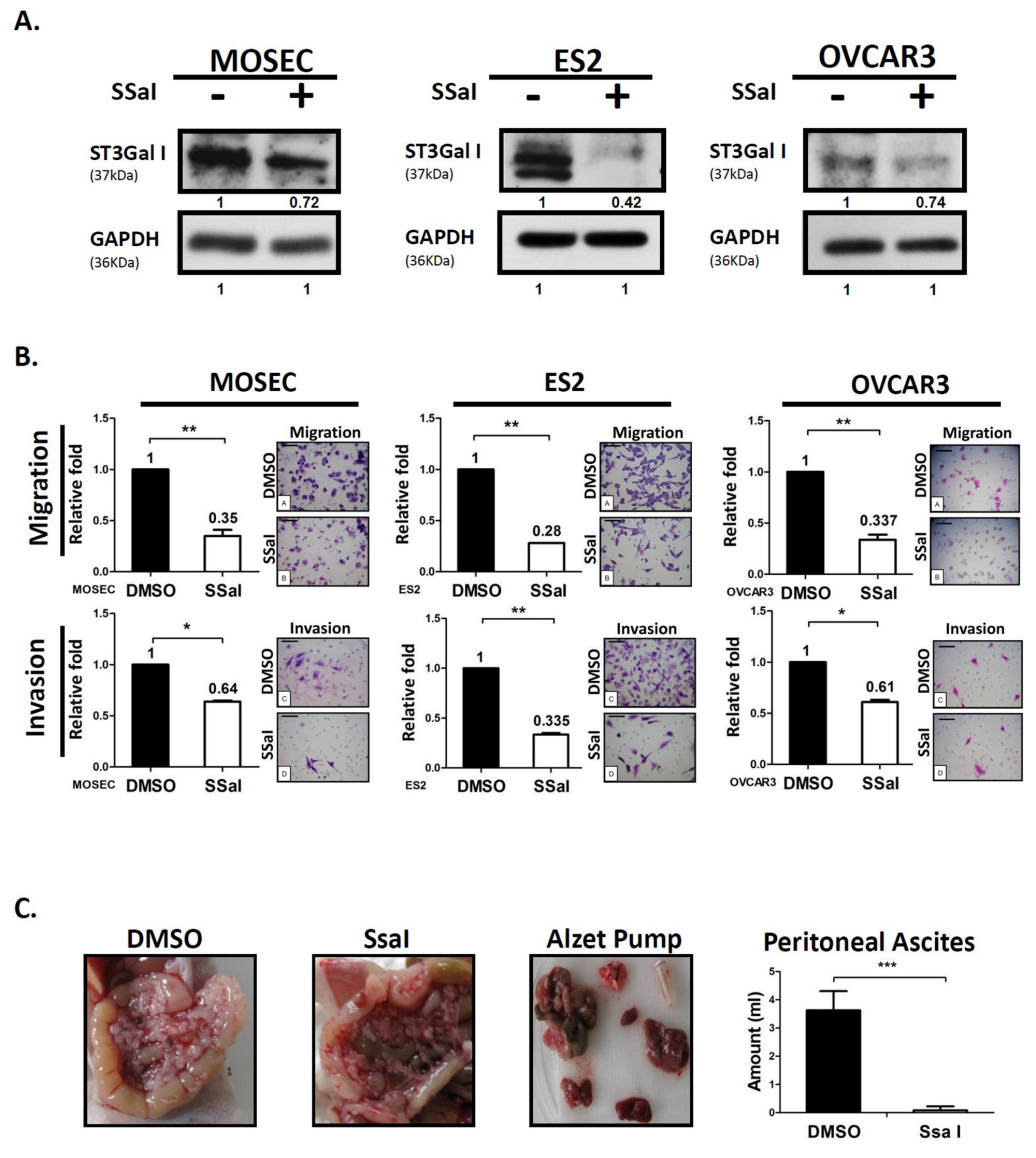
α2,3-sialylation inhibitor SsaI suppresses ovarian cancer tumor migration and peritoneal dissemination **(A)** Quantification of ST3GalI in ovarian cancer cells (MOSEC, ES2, and OVCAR3) treated with 100μM SsaI or DMSO control for 72h by western blot; GAPDH was used as internal control. **(B)** The Transwell migration (upper panel) and invasion assay (low panel) on ovarian cancer cells that were stained with Toluidine blue and Crystal violet solution, respectively. Data shown are the mean ± SD of 3 separate experiments. **(C)** MOSEC cells (2×10^6^) were injected into the peritoneal cavity of 8weekold female C57BL/6 mice (n=10). ALZET Micro-Osmotic Pumps were filled with either 100μM SsaI or DMSO as control was implanted subcutaneously. The mice were sacrificed after 4 weeks and the amount of ascites was measured. The body weights of mice were recorded after injecting MOSEC cells and implantation of pumps implantation. Data shown are the mean ± SD of 5 mice. Peritoneal seeding and carcinomatosis were analyzed in the SsaI and DMSO control groups. (*: *p*< 0.05, **: *p*<0.01, ***: *p*< 0.001.)

Next, we investigated if the α2,3-sialylation inhibitor, SsaI, inhibited peritoneal seeding and carcinomatosis of ovarian cancer and the mechanism by which SsaI modified the behavior of cancer cells. We observed that SsaI treatment down regulated ST3GalI in three ovarian cell lines (MOSEC, ES2, and OVCAR3) compared to the DMSO control (Figure 3A). Further, Transwell assay demonstrated that SsaI treated MOSEC, ES2, and OVCAR3 ovarian cancer cells demonstrated significant inhibition of motility (Figure 3B). These results indicate that ST3GalI may regulate tumor cell motility and hence a potential prognostic marker of human ovarian cancer. More than half of ovarian cancer patients are found in an advanced stage where the ovarian cancer cells have disseminated into the peritoneal cavity to form peritoneal seeding and sometimes carcinomatosis. To investigate this, we injected MOSEC cells into the peritoneal cavity of 8 week-old female C57BL/6 mice along with continuous delivery of either 100μM SsaI or DMSO control from ALZET Micro-Osmotic pumps. We observed peritoneal carcinomatosis with a large amount of ascites in the control mice, mimicking the clinical symptoms of human ovarian cancer (Figure 3C). In contrast, the SsaI-inoculated mice were asymptomatic and demonstrated significantly lower peritoneal carcinomatosis and ascites (Figure 3C). Interestingly, SsaI mildly inhibited cell proliferation in the MOSEC cell line as analyzed by MTT assay (Supplementary Figure 6A), and showed negligible decrease in mouse body weight (Supplementary Figure 6B). Taken together, these data suggested that SsaI, the ST3GalI inhibitor, significantly reduced tumor migration and peritoneal dissemination without significantly affecting growth.

### SsaI inhibits EGFR signaling and shows synergistic effect with TKI

**Figure 4:**
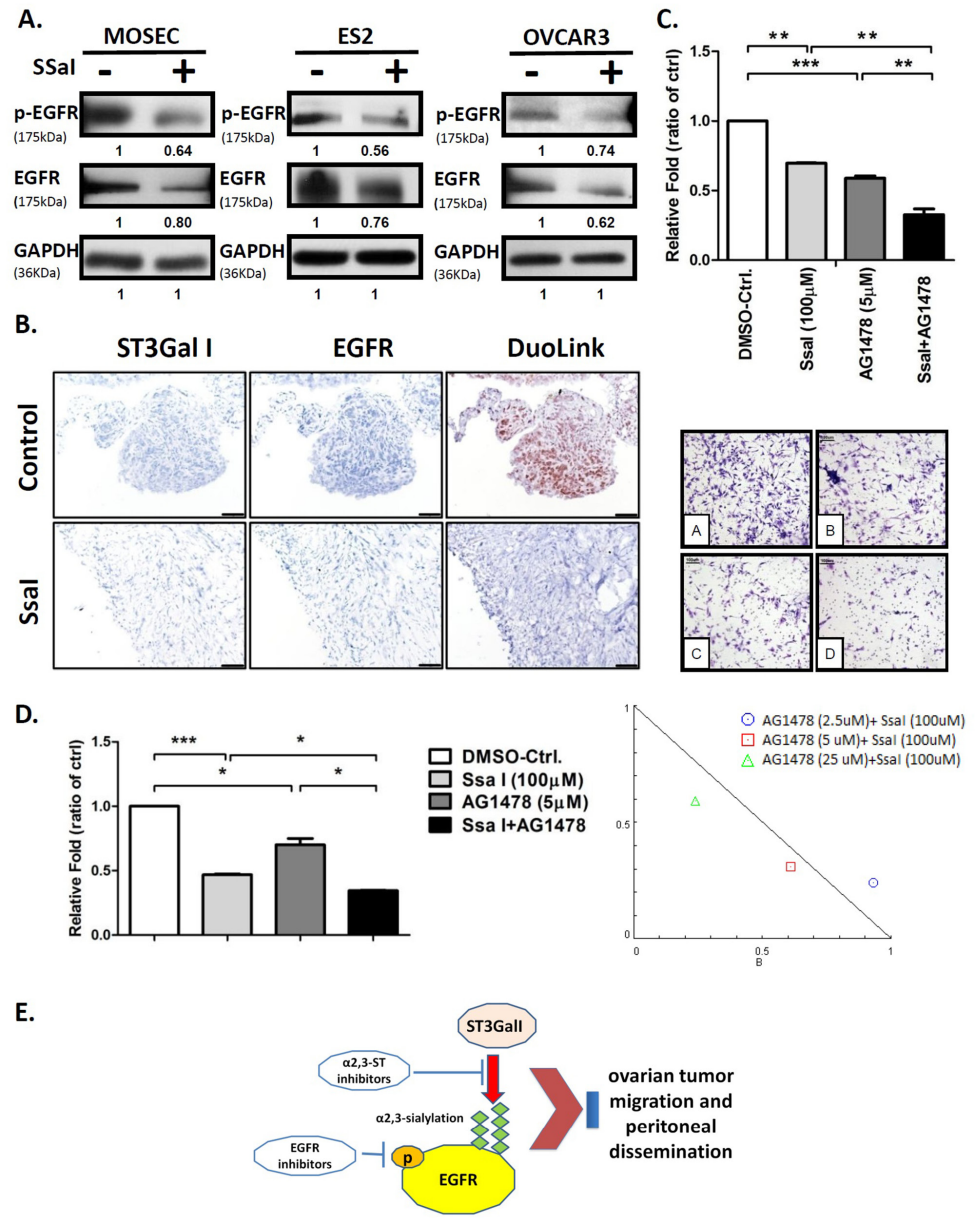
α2,3-sialylation inhibitor SsaI affects EGFR signaling and synergizes with TKI **(A)** Quantification of EGFR and phospho-EGFR in ES2 cells treated with 100μM SSaI or DMSO control for 72h; GAPDH was used as control (same GAPDH as in Figure 3). **(B)** Intraperitoneal tumors from B6 mice treated with either SsaI or DMSO control were stained by the Duolink *in situ* IHC staining kit to analyze for ST3GalI and EGFR. **(C)** ES2 cells were treated with 100μM SsaI and 5μm AG1478 (EGFR inhibitor) were subjected to Transwell matrigel invasion assay. Total numbers of cells were counted in 7 to 10 random fields. Data shown are the mean ± SD of 3 separate experiments. **(D)** Synergy between ST3GalI and EGFR were determined by treating ES2 cells with either SsaI orAG1478 (EGFR inhibitor) or both for 48h followed by determination of their effects on cell proliferation rate. Data shown are the mean ± SD of 3 independent experiments. **(E)** A proposed mechanism of the interaction between ST3GalI and the EGFR signaling pathway.

To examine if SsaI inhibited the EGFR signaling pathway through ST3GalI, we treated the ovarian cancer cell lines with SsaI and found that EGFR expression was down-regulated (Figure 4A). Besides inhibiting the sialylation of EGFR, SsaI interfered with the protein-protein interaction between ST3GalI and EGFR. The intraperitoneal tumor tissues from the B6 mice that were treated with SsaI showed decreased expression of ST3GalI and EGFR compared to the control mice based on DuoLink *in situ* double staining (Figure 4B). This suggested that SsaI suppressed tumor migration and peritoneal dissemination in ovarian cancer via ST3GalI-EGFR signaling.

Further, we investigated the synergy between ST3GalI and EGFR. Towards this, we treated ES2 cells with SsaI in presence or absence of the EGFR inhibitor, AG1478. We observed that cells treated with both SsaI and AG1478 showed significant inhibition of tumor invasion in the Transwell invasion assay compared to cells treated with SsaI alone or the negative control (Figure 4C). Also, we noted that EGF stimulation was suppressed by SsaI treatment in the Transwell matrigel assay (Supplementary Figure 7). Moreover, we demonstrated synergestic effects of the SsaI and AG1478 inhibitor combination over several concentrations (Figure 4D, Table 2). Hence, these data demonstrated that α2,3-sialylation inhibitors may be useful in future ovarian cancer therapy to synergize with TKI (tyrosine kinase inhibitors).

**Table 2:** Synergistic effects of SsaI and EGFR inhibitor

SsaI (μM)	AG1478 (μM)	CI*
100	2.5	1.17
100	5	0.92
100	25	0.84
25	25	0.78
25	50	0.57

* Combination index (CI) is a quantitative measure of the degree of interaction between different drugs. If CI =1, it denotes additivity; if CI > 1, it denotes antagonism; and if CI < 1, it denotes synergism.

## DISCUSSION

Cell surface glycans are induced during carcinogenesis suggesting that they are involved in human cancers [37]. The sialyltransferases (STs) are classified based on the position of attachment of the donor sialic acid to the acceptor as either α2,3 (ST3), α2,6 (ST6) or α2,8 (ST8), and based on their acceptor specificity (for example, ST3GalI, ST3GalII, etc). The STs catalyze transfer of the sialic acid moiety from a cytidine-5′-monophospho-*N*-acyl-neuraminic acid donor (CMP-Neu5Ac) to the various acceptor glycoconjugates terminating in either galactose (Gal), *N-*acetylgalactosamine (GalNAc) or another sialic acid [38, 39].

ST3GalI is specific for α2,3-sialylation of Galß1, 3GalNAc on the O-linked chains of glycoproteins and glycolipids and has an important role in sialyl-Lex/Lea biosynthesis and sialylation of the Thomsen-Friedenreich antigen [40–42]. In previous studies, the expression of ST3GalI was altered in cancers such as colon, bladder, ovary and breast cancer [43]. Videira *et al* found that the overexpression of ST3GalI was associated with the initial oncogenic transformation of bladder [44]. Kudo *et al* showed that ST3GalI was up-regulated in colorectal cancer [45]. Burchell *et al* showed that ST3GalI was elevated in primary breast carcinomas, compared to normal or benign breast tissues [43]. In our previous work, we found that increased ST3GalI expression contributed directly to increased α2,3-linked sialylation in ovarian serous carcinoma [16]. Therefore, in this study we investigated the mechanistic role of ST3GalI in ovarian cancer. Initially, we investigated the relationship between ST3GalI expression, advanced cancer stage and overall survival rate. Using different microarray datasets, we found that ST3GalI was significantly higher in late-stage cancer patients compared to early-stage cases. Furthermore, *in vitro* and *in vivo* studies revealed that ST3GalI down regulation significantly suppressed cancer cell migration and invasion. Conversely, ST3GalI overexpression enhanced the migratory ability and invasiveness of the cancer cells. Taken together, our data demonstrated that ST3GalI played a significant role in ovarian cancer cell migration and peritoneal dissemination and suggested a potential prognostic role.

Since enhanced sialylation was observed in oncogenic transformation, tumor metastasis, and invasion, inhibition of ST may be a potential treatment strategy. A cell permeable ST inhibitor, SsaI, exhibited inhibition of cellular α2,3-sialyltranserase activity [18, 46]. Our previous study showed that SsaI significantly decreased breast cancer cell (MDA-MB-231) migration [19, 20]. In the present study, we demonstrated inhibitory effects of SsaI on migration and invasion of ovarian cancer cell lines, as well as decreased peritoneal tumor ascites in mice. Interestingly, SsaI downregulated the expression of ST3GalIV in breast cancer cells and not ST3GalI and ST3GalIII [19, 20], but inhibited ST3GalI in ovarian cancer cells. This suggested a probable negative feedback regulation of ST3GalI that needs to be investigated along with the ability of SsaI to inhibit different STs in different cancer types.

Studies on non-small—cell lung cancers and others have revealed that several mechanisms like mutation (T709M) of EGFR receptors or activation of alternative signaling pathways or sialylation regulate EGFR function and inhibitor resistance [47–50]. Further, studies found that Asn420 and 579on the glycans prevented ligand-independent dimerization of EGFR [51–53]. Liu *et al* showed that sialylation and fucosylation suppressed dimerization and autophosphorylation of EGFR and EGF-induced lung cancer cell invasion [54]. Yen *et al* showed that sialylation partially suppressed the phosphorylation of EGFR at Y1068, Y1086, and Y1173 in a TKI-resistant lung cancer cell line with L858R/T790M mutations on EGFR and enhanced EGFR sensitivity to TKI [30]. Therefore, in contrast to lung cancer, our data with ovarian cancer cells suggested that sialylation was positively associated with EGFR function. Further, our co-IP data demonstrated that ST3GalI and EGFR interacted with each other (Figure 2F and 2G), and EGFR expression decreased in the ST3GalI knocked-down cell line. Interestingly, ST3GalI also regulated the transcription of EGFR (Figure 2H). These data demonstrated that sialylation in ovarian cancer may be functionally different than in lung cancer.

There are limitations to our study. First, only serous histology was available in the TCGA dataset and small number of case studies in other genomic datasets. Therefore, a larger-scale investigation of different histologic types in ovarian cancer is necessary. Second, previous studies showed that SsaI was a competitive inhibitor of CMP-Neuc5Ac, but, we found that SsaI down-regulated ST3GalI. This implied that SsaI suppressed α2,3-sialylation via a negative feedback regulation that needs to be investigated further. Third, although the results showed that α2,3-sialyltransferases increased EGFR expression in EOC, the effect of sialylation on EGFR structure or conformation was not directly explored. Moreover, the detailed mechanism by which ST3GalI regulated EGFR is not clear in this study. A marked reduction of ST3GalI expression was observed soon after transfection with the ST3GalI knock-down plasmid, whereas EGFR down regulation as a consequence was found to be relatively stable and long-lived. This implied that ST3GalI may regulate factors upstream of EGFR or induce a negative feedback inhibition of EGFR signaling pathway. Fourth, we used only one EGFR inhibitor, AG1478, which is a specific reversible inhibitor of EGFR that selectively inhibits the ligand-induced autophosphorylation of EGFR and downstream signal transduction events [55]. Moreover, this compound has not been evaluated in clinical trials. Other EGFR inhibitors such as gefitinib, erlotinib, lapitinib and anti-EGFR antibodies that are used in cancer therapy need to be considered in combination with ST inhibitors and their efficacy and safety for ovarian cancer patients needs to be evaluated.

In conclusion, our study showed that ST3GalI was a poor prognostic factor in epithelial ovarian cancer (EOC), especially with regard to survival and metastasis. We demonstrated crosstalk between ST3GalI and EGFR that regulated the migration and invasiveness of ovarian cancer cells. Further, our study demonstrated that α2,3-linked sialylation inhibitors such as SsaI in combination with EGFR inhibitors may be potentially future therapeutic targets for EOC treatment.

## MATERIALS AND METHODS

### Cell culture

The human ovarian carcinoma cell line, ES-2, was cultured in McCoy's 5A medium supplemented with 10% FBS and 1% penicillin–streptomycin. The human ovarian carcinoma cell line, OVCAR-3, was cultured in DMEM/F12 medium supplemented with 10% fetal bovine serum (FBS), 1% non-essential amino acids, 1% sodium pyruvate, 1% L-glutamine and 1% penicillin–streptomycin. The mouse ovarian surface epithelial cell line, MOSEC, was cultured in RPMI1640 supplemented with 10% FBS, 1% non-essential amino acid, 1% sodium pyruvate, 1% L-glutamine, 0.1% beta-mercaptoethanol (beta-ME) and 1% penicillin–streptomycin. All the cells were incubated at 37°C and at 5% CO_2_.

### Thiazolyl blue (MTT) assay

To estimate cell survival and proliferation, the ovarian cancer cell lines (5×10^3^) were grown in RPMI1640 medium in 96 well plates supplemented with 5% charcoal-stripped/heat-inactivated FCS for 24h Then, the cells were treated with either DMSO (control) or 100μM SsaI (Soyasaponin I, S9951 Sigma Soyasaponin I Sigma-Aldrich) for 48h. Further, 200μl thiazolyl blue (MTT, 5mg/ml, Sigma-Aldrich) was added into each well with 1ml of medium for 4h at 37°C followed by 2 ml of 0.04N HCl in isopropanol. After thorough mixing by pipetting and 5 min of incubation at room temperature, the absorbance was read at 570nm and subtracted from background absorbance at 600nm. Results were expressed as the mean ± SD of 3 independent experiments.

### Cell migration assay

Transwell cell migration assay was used to estimate the effect of SsaI on the migration of the ovarian tumor cells. The Transwell consisted of an upper and a lower chamber that were separated by a polycarbonate filter of 8μm pore size with a surface diameter of 6.5mm. The ovarian cancer cells (2 × 10^4^) were added to the upper chamber in serum free medium with either DMSO (control) or 100μM SsaI. The lower chamber was filled with 10% FBS that served as a chemoattractant. After incubation at 37°C for 4h, the non-migratory cells from the upper surface of the filter were removed with wet cotton swabs. Then, cells that had migrated to the lower surface of the filters were fixed, stained with DAPI (1.0μg/ml) and then counted from 7 to 10 random fields of view at 400 × magnification. The data were expressed as the average number of cells per field.

### Cell invasion assay

*In vitro* invasiveness of the ovarian cancer cells was assessed by quantifying the number of cells that invaded the Matrigel. Polycarbonate Transwell filters was coated with ~100μg/100μL of diluted Matrigel solution. Then, the cells (2 × 10^4^) were added to the upper chamber with either DMSO or 100μM SsaI and 10% FBS medium was added as a chemoattractant in the lower chamber followed by incubation at 37°C for 24 h. The cells that travelled through the filter were stained and counted from 7 to 10 random fields of view at 400 × magnification.

### SoyasaponinI preparation

SsaI was prepared from a commercial preparation of soybean saponins. The purification process was performed as described with some modifications. Briefly, non-saponin constituents were extracted from the preparation by gently agitating the saponins (4g) in acetone (25ml) at room temperature followed by centrifuging the mixture at 2000xg for 30 min. This acetone extraction was repeated thrice. The residue was then extracted in distilled water thrice and then washed in acetone to facilitate drying. The final purification was carried out on a preparative HPLC C18 column (10×250mm, Phenomenex). HPLC conditions were as follows: solvent A, acetonitrile/TFA (100:0.1, v/v); solvent B, H2O/TFA (100:0.1, v/v). Elution was done with the following mixtures: 40~45% solvent A (40 min), 45~65% solvent B (10 min). The flow rate was set to 3ml/min and the products were detected at 205nm. The soyasaponin I in this study was >99% pure as determined by an analytical HPLC C18 column (4.6mm×250mm, Phenomenex).

### *In vivo* mouse model

We followed the guidelines for animal experimentation adopted by the Animal Subjects Program (ASP) at Taipei Veterans General Hospital (VGH-TPE). At the day of implantation, MOSEC cells (2×10^6^) were injected into the abdominal/peritoneal cavity of 8 week old female C57BL/6 mice (National Laboratory Animal Center, Taipei, Taiwan). Alzet osmotic miniature pumps (model 1004; DURECT Corporation Charles River Laboratories, L’Arbresle, France) with constant delivery rates of 0.11 l/h for about 4 weeks were filled with either 100μl SsaI in PBS or DMSO as control. The pump had a delayed starting time of approximately 2 days after filling. The osmotic mini-pumps filled with either SsaI or DMSO were implanted under the skin of the mice under sterile conditions and started when no cartilage destruction was detectable (days 0-4). Since the reagents circulated for a few days after the pump was empty, an experimental setting of 28 days was selected.

### Gene transfection

The lentiviral plasmid vectors harboring specific short hairpin RNA (shRNA) sequences for human ST3GalI and a non-target scrambled control were purchased from the RNAi Core Facility (Academia Sinica, Taiwan). The ST3GalI shRNA sequence was 5′-GCGGGAGAAGAAGCCCAATAA-3′(pLKO-TRC005 vector, Clone ID: TRCN0000231839). The ST3GalI shRNA or control plasmids (pLKO-TRC005 vector) were transduced into ovarian cancer cells with the lentivirus package plasmids using Lipofectamine 2000 (Invitrogen Life Technologies, Carlsbad, CA, USA) according to the manufacturer's instructions. Stable transfectants were selected in presence of 1*μ*g/ml puromycin (Sigma-Aldrich, St Louis, MO, USA). For gain of function experiments, human ST3GalI expression plasmid (Origene, RC217696) and control plasmid (empty vector) was transfected using the GenJet plus *in vitro* DNA transfection kit (SignaGen, Maryland, USA).

### RNA isolation and real-time quantitative PCR (RT-qPCR)

The ovarian cancer cells were lysed by TRIzol and centrifugation at 12,000 x g for 10 minutes after adding bromochloropropane (Sigma-Aldrich). Total RNA was precipitated with isopropanol, followed by washing with ethanol to remove impurities and finally dissolved in RNAase-free water (Invitrogen). Total RNA was quantified spectrophotometrically using A260/A280 values. The cDNA synthesis was performed using SuperScript™ II reverse transcriptase (Invitrogen) according to the manufacturer's instructions. The 12μL volume of one reaction with oligo(dT)_12-18_ (500μg/mL), 1 μL dNTP Mix (10 mM each), 1 ng to 5 μg total RNA and sterile distilled water to perform 65°C for 5 min and quick chill on ice. Add 7μL volume of buffer mix with 5X First-Strand Buffer, 0.1 M DTT and RNaseOUT™ (40 units/μL) to perform 42°C for 2 min. Add 1 μL (200 units) of SuperScript™ II RT to perform 42°C for 50 min and 70°C for 15 min. The PCR primers and probes designed using the Roche Online Assay Design Centre for the ST3GalI and EGFR genes and control 18S RNA were as follows: ST3GalI, Forward 5′- TGGTCCTGGAGCTCTCCGAGAA-3′, Reverse 5′- GACTGTCTATCTCAGGCCCATAAGAAGA-3′; EGFR, Forward 5′- ACTCATGCTCTACAACCC-3′, Reverse 5′- CCAATACCTATTCCGTTACAC-3′; and 18S, Forward 5′-GACCAGAGCGAAAAGCAT-3′, Reverse 5′-TCGGAACTACGACGGTATC-3′. The cDNA samples were amplified by real-time PCR using a LightCycler 480 Instrument (Roche). The 20μL volume of one reaction with 10ng/μL cDNA, 50nM Forward and Reverse primer, 2xSYBR reagent and sterile distilled water to perform PCR amplification and Melting curve program. Quantification cycle values (crossing point, Cp) for each gene were calculated as the cycle number at which the reporter fluorescence passed a fixed threshold above the base line. The expression levels of the genes of interest, ST3GalI and EGFR, were normalized in relation to the housekeeping control (18S). All experiments were performed thrice.

### Protein isolation and western blot analysis

For western blot analysis, ovarian cancer cells that were treated with or without SsaI were lysed in a buffer containing 1% Triton X-100 in PBS and protease inhibitor mixture tablets (Roche, Barcelona, Spain). Following 30 min incubation on ice with vortexing, lysed cells were centrifuged (14000 rpm, 20 min, 4°C) to pellet cell debris, and protein concentrations of the resulting cell supernatants were determined by the Bradford Assay (Bio-Rad, Hemel Hempstead, UK), relative to a standard curve prepared from serial dilutions of bovine serum albumin (0–4mg/mL) with absorbance readings at 595nm. 100μg total protein was diluted in equal volumes of 2x protein sample buffer (125mM Tris-HCl (pH 6.8), 4% SDS, 0.02% bromophenol blue, 0.2M DTT, 20% glycerol) was electrophoresed on a 10% ~ 15% SDS-PAGE run 100 voltage, 3hrs and transferred onto Immobilon polyvinylidene difluoride membranes (Millipore, Bedford, MA) 90 voltage, 2hrs. Then, the membranes were incubated 30mins at room temperature in blocking solution (5% nonfat dry milk/0.1% Tween-20/1xPBS) followed by incubating the membranes overnight with the appropriate primary antibodies namely, ST3GalI (Abcam, ab96129, 1:500), EGFR (CST, #4267, 1:1000) or phospho-EGFR (CST, #2234, 1:1000). Then, the membranes were washed 10min trice with 0.05% Tween-20/PBS followed by incubation 2hrs at room temperature with anti-Rabbit HRP-conjugated secondary antibody (Geneisland, #G701120, 1:5000). The blots were developed with enhanced chemiluminescence reagent (Amersham Pharmacia Biotech) followed by exposure to X-ray film to visualize the protein bands. The protein bands were quantitated using ImageJ analysis by determining the relative intensity for each experimental band by normalizing its absolute intensity to that of the control.

### Immunohistochemistry

IHC specimens (59) from the CJ2 human ovarian cancer tissue array were obtained from a commercial tissue array (Super Bio Chips, Seoul, S. Korea) with complete clinical data, including clinical stage, grade and overall survival (OS). Tissue sections were immersed in a coplin jar filled with 1x Dako target retrieval solution (Dako, Denmark; Code S1699), heated for 5 min at 121°C and then cooled to 85°C. Then, the endogenous peroxidase activity was quenched by treating the specimens with 0.6% hydrogen peroxide/methanol mix. The sections were further blocked with goat serum (BioGenex, San Ramon, CA, USA) for 30 min at room temperature to prevent non-specific binding. Then, the sections were incubated overnight with anti-ST3GalI Ab (Abcam, ab96129, 1:50) at 4°C followed by staining with AEC Substrate-Chromogen (DAKO, Denmark) and counterstained with hematoxylin. For the negative control, sections were treated with PBS instead of the antibody. The IHC staining was quantified based on the staining intensity score (0, no staining; 1, weak; 2, moderate; 3, strong). A strong intensity score indicated high expression of ST3GalI, whereas, other intensity scores indicated different grades of lower expression.

### Duolink *In Situ* staining

Protein–protein interactions were detected using a Duolink *In Situ* Kit (Olink Bioscience, Uppsala, Sweden; PLA probe anti-rabbit plus; PLA probe anti-mouse minus; Detection Kit orange). In brief, tissue specimens from the *in vivo* mouse experiment were incubated with primary antibodies against ST3GalI and EGFR for 2h at room temperature. Then, the slides were washed twice in suitable buffer 1x wash buffer A (0.01 M Tris, 0.15 M NaCl and 0.05% Tween 20) for 5 min followed by incubation with the 2 PLA probes (1:5 in appropriate buffer antibody dilution) for 60 min at 37°C. Then, the specimens were incubated with ligase (1:40 in buffer high purity water). After washing twice in 1x wash buffer A for 2 min, the amplification procedure was carried out with the polymerase (1:80) for 100 min at 37°C. Then, the specimens were washed twice in 1x Wash Buffer B for 10 min and 0.01x Wash Buffer B for 1 min. The slides were finally incubated for 15 min with Oregon green phalloidin antibody (1:50, Invitrogen) at room temperature and visualized under a light microscope (Nikon, Ci).

### Analysis of ovarian cancer and prognosis in the TCGA project

The clinical and protein expression data of ovarian cancer (high-grade serous cystadenocarcinoma) from the *TCGA* were downloaded from the Oncomine website (http://www.oncomine.org/). The patients in the ovarian serous cystadenocarcinoma dataset (Human Genome U133A array, 12,624 measured genes, 562 cases) were categorized using a total of 20 STs including α2,3-, α2,6-, and α2,8-sialyltransferases with a tercile approach. The patients were categorized as high or low expressing if the mRNA of interest was in the upper one-third or in the lower two-thirds, respectively. We analyzed the disease free survival (DFS), overall survival (OS) and the correlation between ST3GalI and EGFR using this dataset. Furthermore, different datasets (Schwartz ovarian, HumanGeneFL array [56]; Hendrix ovarian, Human Genome U133A array [57]; Denkart ovarian, Human Genome U133A array [58]; Lu ovarian, Human Genome U95 array, [59]) from the Oncomine website were used to investigate if ST3GalI regulated ovarian cancer dissemination. Further, the patients were sub-divided into either early stage (stages 1 and 2) or late stage (stages 3 and 4) and the expression of ST3GalI was compared between these 2 groups.

### Microarray analysis

The microarray experiments were conducted using the L1000 Operating Procedure (L1000 SOP). In brief, a human ovarian cancer cell line, ES2, was grown in commercial 96-well plate for one day and the cells lysed with lysis buffer (L1000 kit) for 30 mins. The lysate was stored at 80°C overnight and then transferred to a 384-well plate for the L1000 assay that was conducted according to the commercial protocol (http://s3.amazonaws.com/support.lincscloud.org/protocols/data_generation/L1000_SOP.pdf). Gene expression profiles in the control and study group were detected by L1000 array technology. Up and down probe sets were selected by 2-sample t-test with a *p* value lower than 0.01 and fold change greater than 1.5-fold considered as significant. The up and down probe sets were input into GeneE software to run the analysis and interpret the transcription profile data. The expression of sialylation and significant cell receptors, including EGFR, IGFR, VEGFR and PDGFR was determined.

### Analysis of CCLE ovarian cancer and drug efficiency

The mRNA expression of ST3GalI-VI and the drug efficiency (Actarea) were derived from the cancer cell line encyclopedia (CCLE) ovarian cancer dataset (https://portals.broadinstitute.org/ccle/home). The TKI drugs included erlotinib, lapatinib, ZD-6474, and TKI258.

### *In Vitro* cell viability assays

To assay the synergy between ST3GalI and EGFR, ES2 cells were treated with 100μM SsaI in presence or absence of EGFR inhibitor (Sigma, AG1478) in 96-well plates (4×10^3^ per well) and cultured for 48h. Then, the cells were collected and counted by staining a sample with trypan blue and the cell proliferation was measured with the MTT assay. Following 20μl of a sterile, filtered 3-(4, 5-dimethylthiazol-2-yl)-2, 5-diphenyltetrazolium bromide (MTT) solution (5 mg/ml) in 1xPBS (pH 7.4) was added to each well and incubated for 4 hrs at 37 °C, and adding 150 μL dimethyl sulfoxide (DMSO) and incubating 10mins at 37 °C. Absorbance was read at 560 nm on a microplate reader. The results were analyzed using the CompuSyn software (http://www.combosyn.com/). The IC50 of each drug was determined by interpolation from the dose–response curves. The resulting combination index (CI) determined the quantitative degree of interaction between different drugs (CI=1 was denoted additivity; CI>1 was denoted antagonism; CI<1 was denoted synergism). For interpretation, the combination was plotted as Log_10_CI versus Fa (fraction affected defined as 1 – survival fraction). Based on these plots, additivity was defined as logCI = 0; synergy was defined as log_10_CIN<0; antagonism was defined as log_10_CIN>0.

### Immunoprecipitation (IP)

Immunoprecipitation of ST3GalI and EGFR was performed using a Pierce Crosslink IP kit (#26147) according to the manufacturer's protocol. In brief, 10μg of Erbitux (Merck, 2mg/mL) antibody was coupled and cross-linked to protein A/G plus agarose resin (0.55mL of settled resin supplied as a 50% slurry). 1×10^8^ cells ES2-shST3Gal1 and ES2-scramble infected cells were rinsed with PBS, scraped and incubated in lysis buffer (0.025M Tris, 0.15M NaCl, 1mM EDTA, 1% NP40 and 5% glycerol supplemented with 1× EDTA-free protease inhibitor, Roche) for 1h at 4°C. The cell lysate was centrifuged at 10,000xg for 15min. The protein concentration in the supernatant was quantified (Bio-rad protein assay). Then, 1mg total protein was incubated with the cross-linked antibody overnight at 4°C. The immunoprecipitated proteins were washed trice with 200μL IP Lysis/Wash Buffer (0.025M Tris, 0.15M NaCl, 0.001M EDTA, 1% NP-40, 5% glycerol; pH 7.4), eluted with 60μL elution buffer (pH 2.8, contains primary amine) form protein A/G plus agarose resin binding identify EGFR protein column and resolved with 5x non-reducing lane marker sample Buffer (0.3M Tris-HCl, 5% SDS, 50% glycerol, lane marker tracking dye; pH 6.8) on a 12% SDS–PAGE. Then, western blot analysis was performed to detect either EGFR or ST3GalI using the appropriate antibodies previously mentioned (western blot section of methods).

### Statistical analysis

Statistical analysis was performed using the SPSS software program. For cell invasion assay data, non-paired Student's t-test was used for comparisons between groups. The Kaplan-Meier survival curves were plotted for ovarian cancer patients and the *P* values were determined using the log-rank test for censored survival data. Survival time was censored if the patient was alive at the time of evaluation. The relationship between IHC expression and other clinical or tumor parameters was calculated using the χ2 test. A *P* <0.05 was considered significant.

## SUPPLEMENTARY MATERIALS FIGURES AND TABLES



## Abbreviations

EOC: Epithelial ovarian cancer
EGFR: Epidermal growth factor receptor
SsaI: soyasaponin I
OS: overall survival
ST: sialyltransferase
