# Supplementary material for: α2,3-sialyltransferase type I regulates migration and peritoneal dissemination of ovarian cancer cells
Source: Oncotarget. 2017 Mar 7;8(17):29013–27. doi: 10.18632/oncotarget.15994 (PMC5438708; doi:10.18632/oncotarget.15994)
Supplement: Supplementary file 1 [file oncotarget-08-29013-s001.pdf]

α2,3-sialyltransferase type I regulates migration and peritoneal dissemination of ovarian cancer cells

SUPPLEMENTARY FIGURES

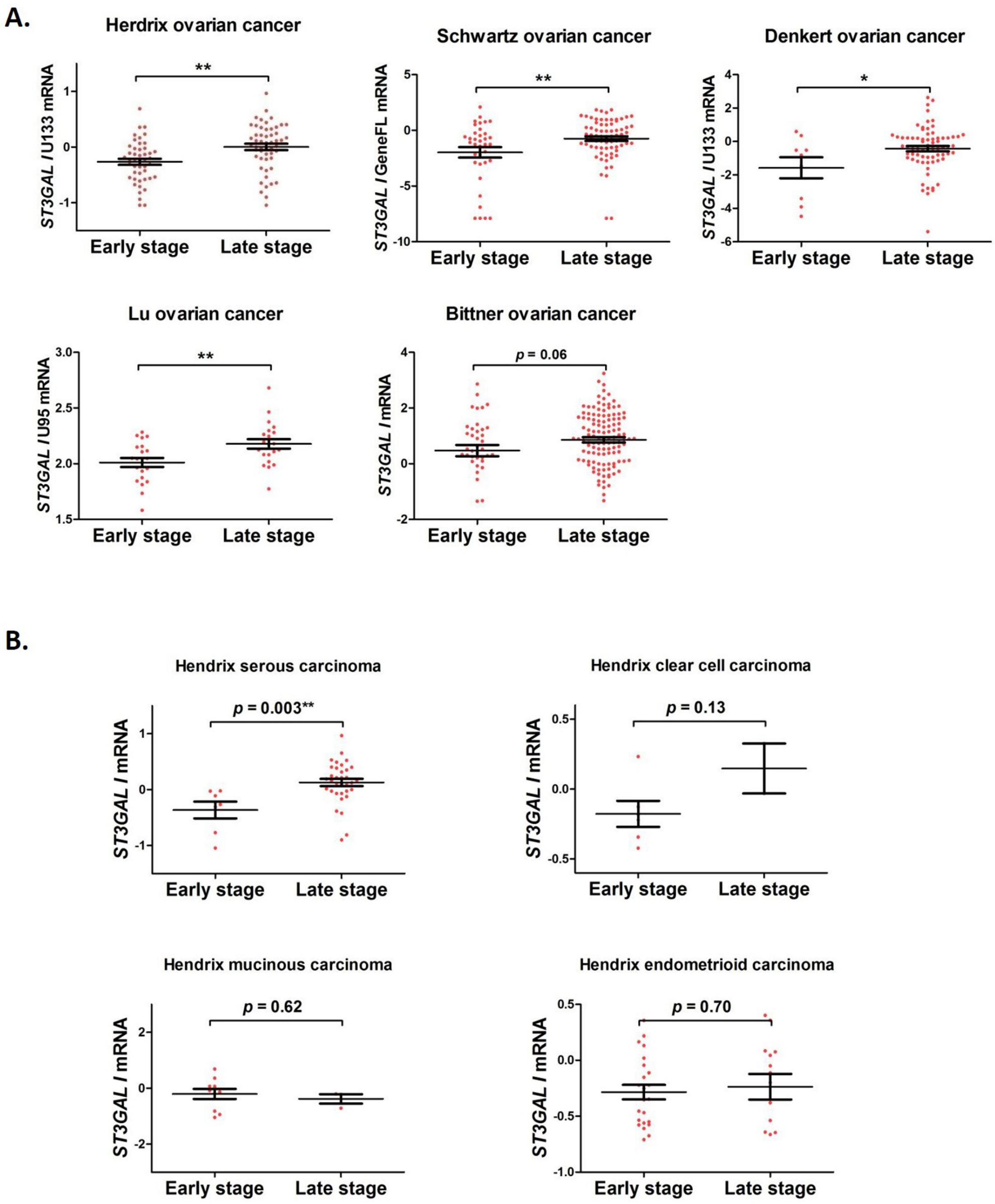

Supplementary Figure 1: The expression of ST3GalI predicts advanced stage of ovarian cancer. (A) Different ovarian cancer datasets (Schwartz, Hendrix, Denker, Lu, and Bittner ovarian cancer) from the Oncomine website were investigated for ST3GalI association with ovarian cancer dissemination. The patients were divided into early-stage (stages 1 and 2) or late-stage (stages 3 and 4) groups. The mRNA levels of ST3GalI was compared between these 2 groups (*: $p<0.05$, **: $p<0.01$). **(B)** Comparison of ST3GalI mRNA expression between different subtypes of EOC, including serous, clear cell, endometrioid, and mucinous.

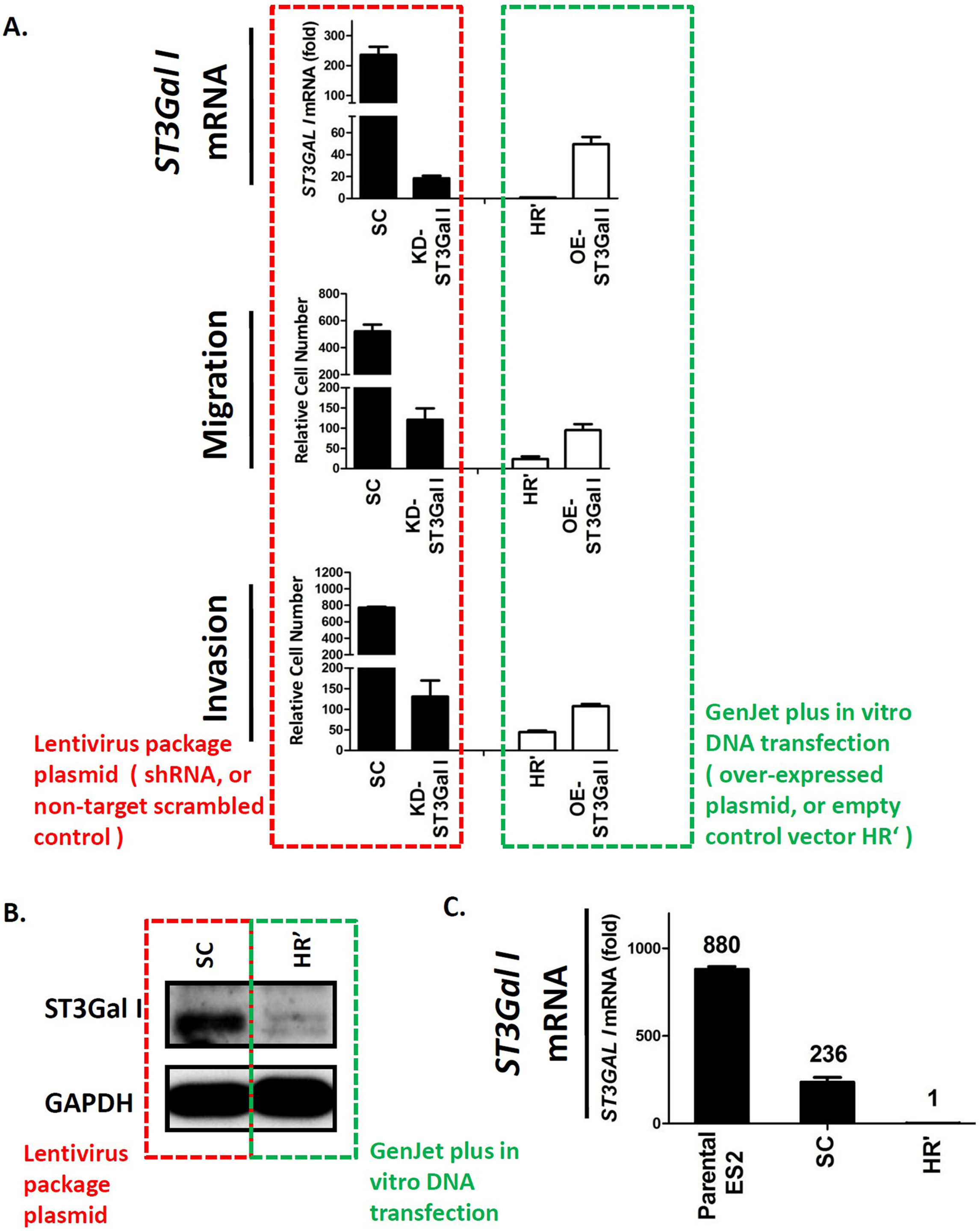

Supplementary Figure 2: Effect of ST3GalI down regulation or overexpression on migration of ovarian cancer cells (A) The human ovarian cancer cell, ES2, in presence or absence of ST3GalI knockdown or over-expression were subjected to Transwell migration and matrigel invasion assay. Total number of cells in 7 random fields were counted and analyzed. Data shown are the mean ± SD of 3 independent experiments (*: $p<0.05$; **: $p<0.01$). Cells that were subject to migration and invasion assay were also used for RNA analysis. The red dotted line represents the shRNA or non-target scrambled control cells transfected by lentivirus package plasmid. The green dotted line represented the ST3GalI overexpressing or empty vector (HR') cells transfected by GenJet plus *in vitro* DNA transfection kit. **(B)** Western blot analysis of ST3GalI in SC and HR' cells; GAPDH was used as control. **(C)** RNA expression of SC, HR', and parental ovarian cancer cell ES2; 18s was used as internal control.

A.

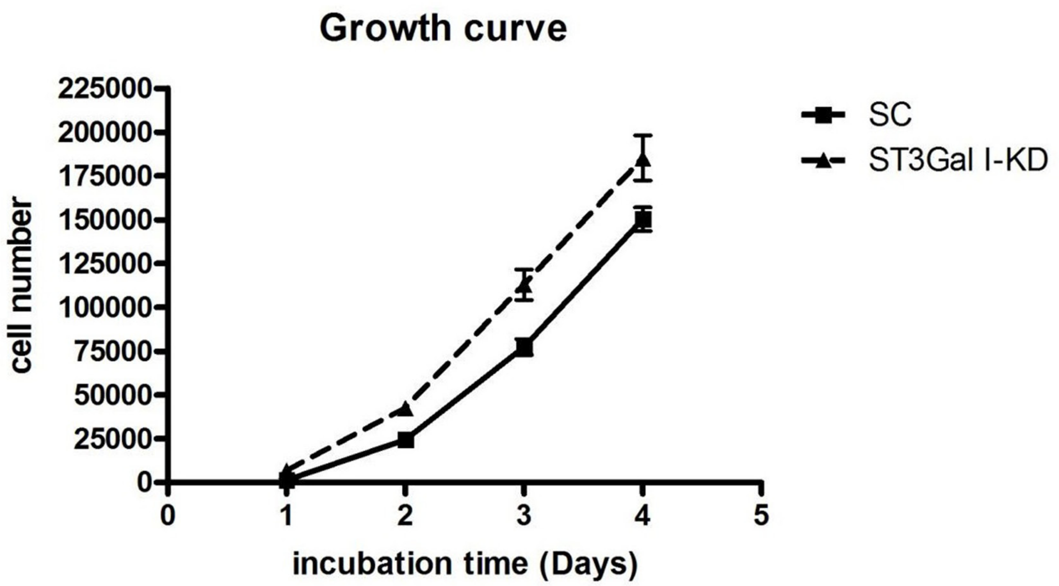

B.

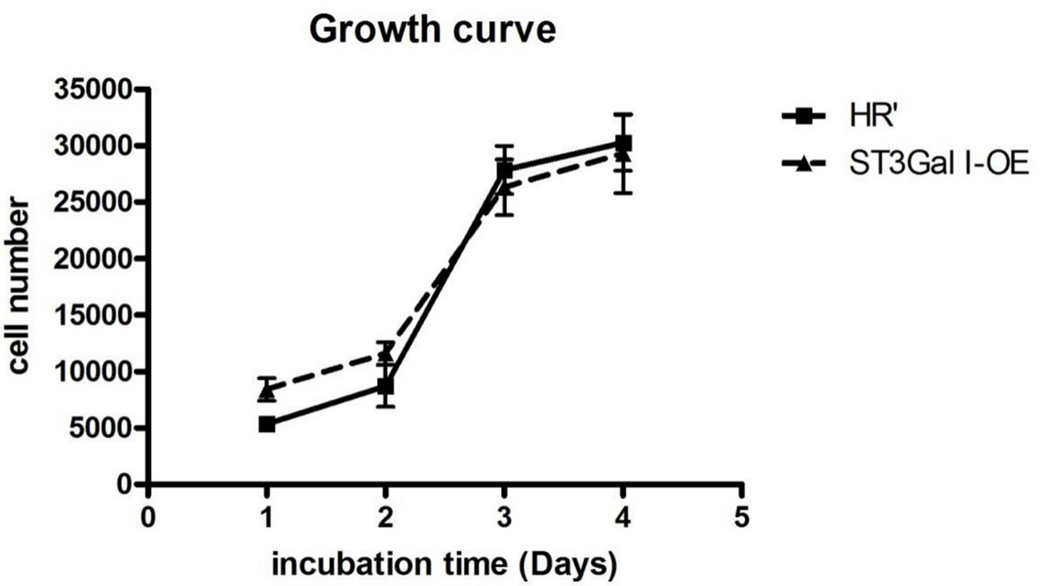

Supplementary Figure 3: Effects of ST3GalI on ovarian cancer cell growth. **(A-B)** Growth curve of ST3GalI knocked down or overexpressed cells were determined with appropriate controls to determine the effects of ST3GalI on ovarian cancer cell growth. Data shown are the mean ± SD of 5 separate samples.

A.

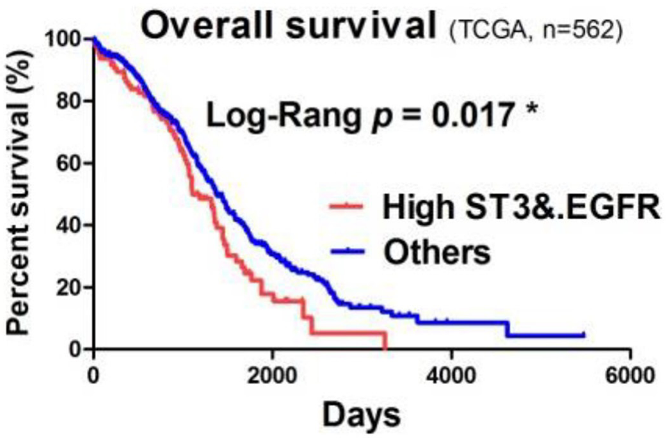

B.

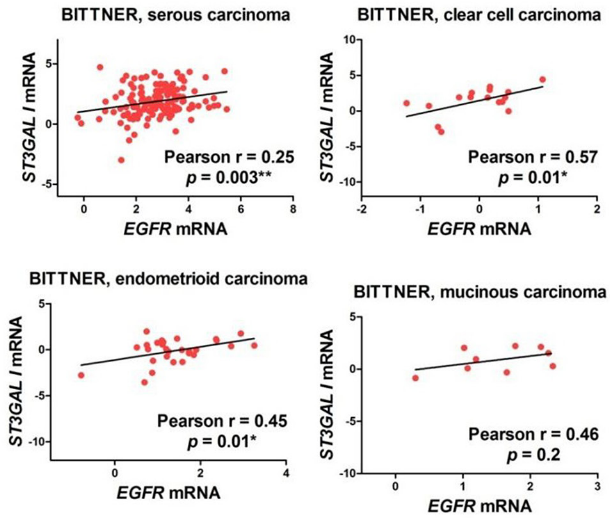

C.

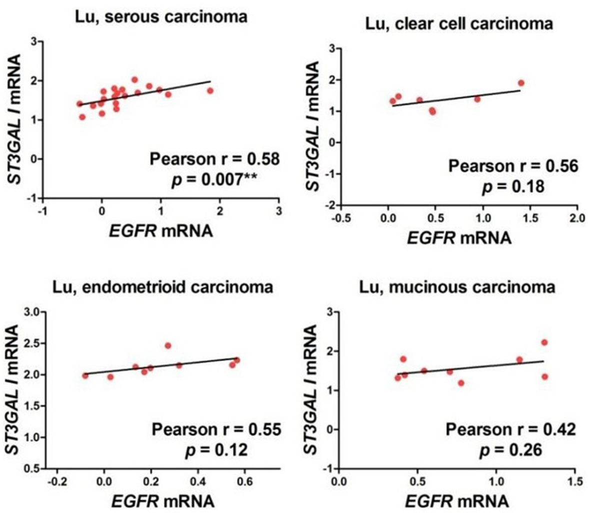

Supplementary Figure 4: The correlation between ST3GalI and EGFR in subtypes of EOC. **(A)** OS of patients with simultaneous high ST3GalI and EGFR levels were compared to the other cases in TCGA ovarian cancer genomics. **(B-C)** Association of ST3GalI and EGFR in different subtypes of EOC, including serous, clear cell, endometrioid, and mucinous that were derived from 2 datasets was determined by analyzing their RNA expression.

A.

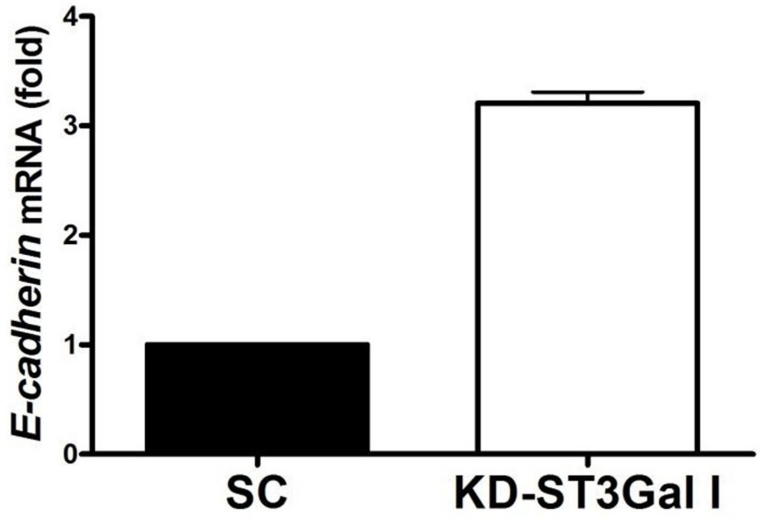

B.

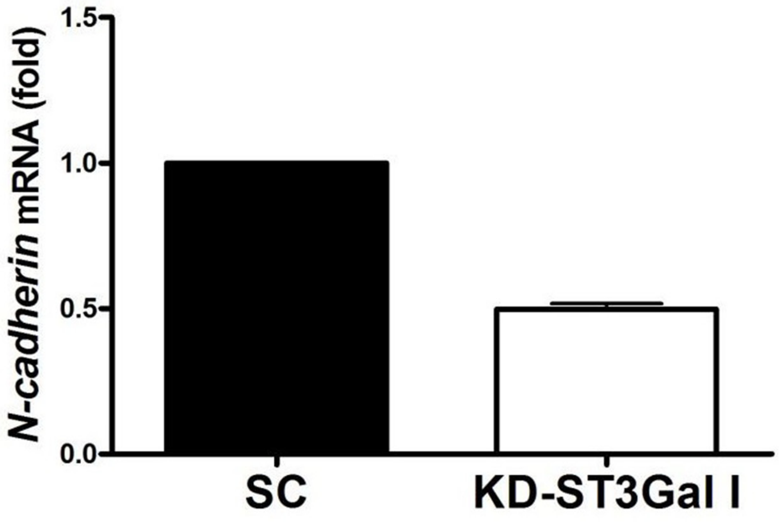

Supplementary Figure 5: The expression of EMT factors ST3GalI knockdown cells. The RNA expression of epithelial-mesenchymal transition (EMT) factors, including E-cadherin and N-cadherin in cells with downregulated ST3GalI; 18S was used as an internal control.

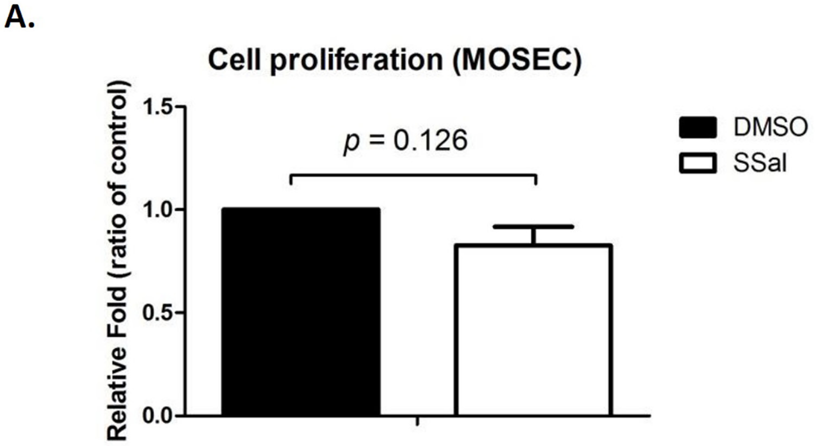

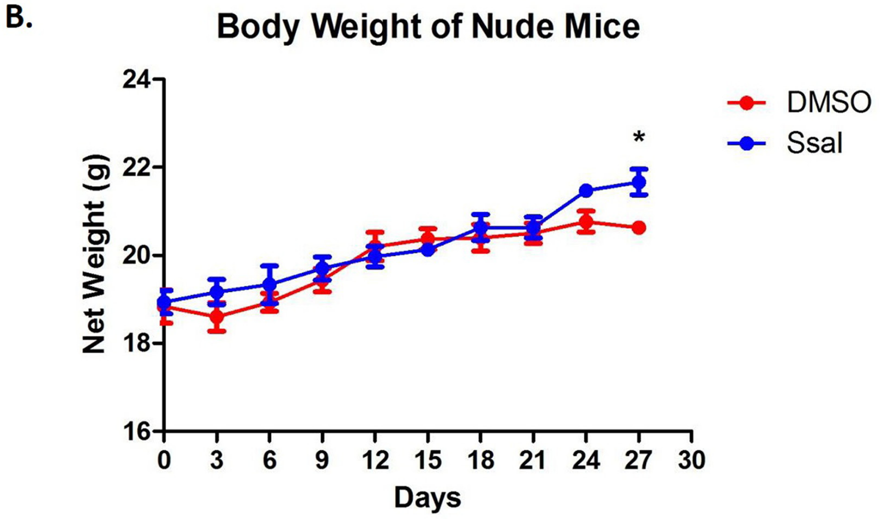

Supplementary Figure 6: α2,3-sialylation inhibitor SsaI does not significantly inhibit tumor growth. **(A)** Cell proliferation of MOSEC, ES2, and OVCAR3 ovarian cancer cells treated with SsaI or a DMSO control for 48h was determined by MTT assay. Data shown are the mean ± SD of 3 independent experiments. **(B)** Body weight analysis of mice injected with MOSEC cells and treated with either SsaI or DMSO (*: $p<0.05$).

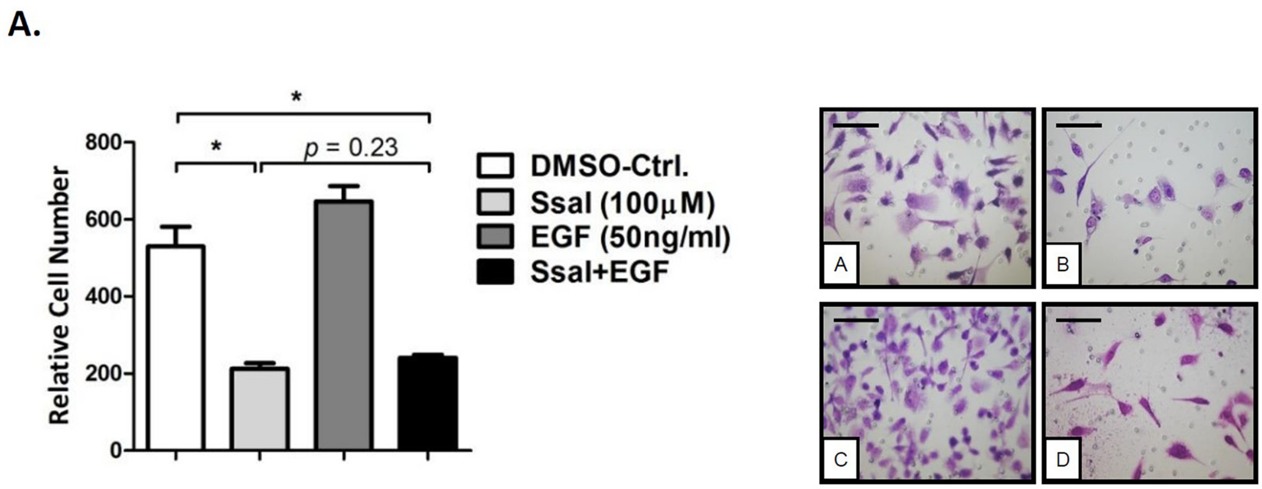

Supplementary Figure 7: α2,3-sialylation inhibitor SsaI affects EGFR signaling and shows synergy with TKI. ES2 cells treated with either 100μM SsaI or 50ng/ml EGF or both were subjected to Transwell matrigel invasion assay and total numbers of cells were counted in 7 to 10 random fields. Data shown are the mean ± SD of 3 independent experiments.
